# Cauda Equina Syndrome: A Narrative Review Exploring the Pathophysiology, Clinical Spectrum, Diagnostic Strategies, and Time-Critical Management

**DOI:** 10.7759/cureus.108930

**Published:** 2026-05-15

**Authors:** Tharun T Aduri, Pankaj K Mishra, Virendra Verma, Naman Jain, Swasti S Prasad

**Affiliations:** 1 Department of Orthopaedics, All India Institute of Medical Sciences, Bhopal, IND; 2 Department of orthopaedics, All India Institute of Medical Sciences, Bhopal, IND

**Keywords:** bladder dysfunction, cauda equina syndrome, lumbar disc herniation, neuroprotection, spinal emergency, surgical timing

## Abstract

Cauda equina syndrome (CES) is an uncommon but severe neurological condition resulting from compression of the lumbosacral nerve roots, leading to motor, sensory, and autonomic dysfunction. Despite its low incidence, CES carries a high risk of permanent disability and medico-legal consequences due to the narrow therapeutic window for intervention. This narrative review synthesizes current evidence on the anatomical basis, pathophysiology, etiology, clinical presentation, diagnostic pathways, classification systems, and management strategies for CES. Particular emphasis is placed on the prognostic distinction between incomplete CES (CES-I) and CES with urinary retention (CES-R), as well as the ongoing debate surrounding the optimal timing of surgical decompression. Long-term outcomes, rehabilitation principles, and emerging neuroprotective strategies are also discussed. Early recognition, prompt imaging, and urgent decompression remain central to improving neurological recovery and quality of life in affected patients.

## Introduction and background

Cauda equina syndrome (CES) is an uncommon yet potentially catastrophic neurological disorder caused by compression of the lumbosacral nerve roots within the spinal canal, most often occurring distal to the conus medullaris at the L1 to L2 level [1]. It is characterized by a combination of lower motor neuron deficits and autonomic disturbances, classically including bilateral radicular pain, saddle anesthesia, bladder and bowel dysfunction, and varying degrees of motor weakness or reflex loss in the lower extremities [2]. Due to the narrow window of opportunity to prevent irreversible neurological damage, CES represents one of the few true neurosurgical emergencies in spinal practice.

The annual incidence of CES is estimated at approximately one to three cases per 100,000 population. Although it accounts for fewer than 2% of all lumbar disc herniations, it is associated with a disproportionately high number of medico-legal claims related to spinal disorders [3,4]. From a pathophysiological perspective, CES results from a combination of direct mechanical compression and ischemic impairment of the cauda equina nerve roots, leading to axonal injury and demyelination. Delayed decompression substantially increases the risk of permanent autonomic dysfunction, highlighting the urgency of early intervention [5].

The association between intervertebral disc rupture and cauda equina compression was first described by Mixter and Barr in 1934, a landmark observation that shaped modern spinal surgery and neurology [6]. Since then, diagnostic approaches have evolved from reliance on clinical symptomatology to advanced imaging-based evaluation. The introduction of MRI in the late 20th century revolutionized CES diagnosis by enabling rapid and accurate visualization of compressive pathology, thereby facilitating timely surgical decision making [7]. Earlier modalities, such as myelography and CT, while useful, lacked sufficient soft tissue resolution and often contributed to diagnostic delay. In recent decades, management strategies have shifted from symptom-based recognition to a time-sensitive treatment paradigm, with increasing emphasis on early surgical decompression, often within 24 hours, to maximize neurological recovery. Despite this progress, significant debate remains regarding the precise definition, diagnostic thresholds, and prognostic classification of CES [8].

This narrative review synthesizes contemporary evidence on the etiology, clinical features, diagnostic pathways, and management principles of CES. By integrating historical context with modern imaging techniques, classification systems, and outcome data, it aims to address ongoing controversies, particularly those surrounding the optimal timing of decompression and the prognostic distinction between incomplete CES (CES-I) and CES with retention (CES-R) [9]. In addition, long-term rehabilitation strategies and quality-of-life outcomes are examined, highlighting the importance of early recognition, multidisciplinary care, and emerging neuroprotective therapies.

## Review

Methods

A narrative literature review was conducted involving peer-reviewed articles, systematic reviews, landmark cohort studies, and clinical guidelines related to CES. Relevant literature was identified through searches of PubMed, MEDLINE, and Google Scholar databases for studies published predominantly in the English language between January 2000 and December 2025, with selective inclusion of landmark older studies considered historically or clinically relevant, focusing on the anatomy, pathophysiology, clinical presentation, diagnostic evaluation, classification systems, surgical timing, outcomes, and rehabilitation of CES. Search terms included combinations of “cauda equina syndrome,” “lumbar disc herniation,” “conus medullaris syndrome,” “surgical decompression,” “urinary retention,” and “spinal emergency,” used in various combinations to refine the search strategy.

Eligible articles included narrative reviews, systematic reviews, meta-analyses, prospective and retrospective cohort studies, case control studies, clinical guidelines, and landmark observational studies relevant to the diagnosis and management of CES. Case reports with limited clinical applicability, non-English publications without accessible translations, duplicate studies, and studies lacking adequate methodological clarity were excluded when appropriate.

Articles were selected based on their clinical relevance, methodological quality, contemporary applicability, and contribution to understanding CES. Given the narrative nature of this review, formal PRISMA (Preferred Reporting Items for Systematic reviews and Meta-Analyses) methodology, quantitative meta-analysis, and structured risk-of-bias assessment were not performed. Instead, the objective was to synthesize current evidence into a clinically practical and educational review for spine surgeons and clinicians involved in diagnosing and managing CES.

Results

The cauda equina, literally meaning “horse’s tail,” comprises the bundle of lumbosacral nerve roots extending inferiorly from the conus medullaris, which in adults typically terminates at the L1-L2 vertebral level. These roots, spanning segments L2 to S5, descend within the lumbar cistern inside the dural sac before exiting through their respective intervertebral foramina to innervate the lower limbs, perineum, and pelvic viscera [10]. Although anatomically central, they function physiologically as peripheral nerves, forming a critical interface between the central and peripheral nervous systems. Each nerve root contains both motor and sensory fibers, while the sacral roots (S2-S4) provide parasympathetic innervation to the bladder, bowel, and sexual organs. This anatomical organization accounts for the hallmark autonomic and perineal sensory disturbances observed in CES [11].

The vascular supply of the cauda equina is derived primarily from radicular branches of the lumbar and lateral sacral arteries, with supplementary contribution from distal branches of the anterior spinal artery [12]. Parke and colleagues demonstrated that the distal nerve roots are particularly susceptible to ischemia due to their terminal-type vascular pattern [13]. Consequently, sustained compression compromises both neural integrity and perfusion, a central mechanism underlying CES pathogenesis.

CES pathophysiology is multifactorial, with mechanical compression of the nerve roots initiating a cascade of secondary ischemic injury. Neural deformation disrupts axonal conduction, impairs microcirculation, and triggers venous congestion and arterial insufficiency [14]. Experimental and clinical data suggest that irreversible neuronal injury may occur when compression persists beyond 6-12 hours [15]. CES is therefore best conceptualized as a neuro-ischemic syndrome in which both the severity and duration of compression determine neurological outcome.

Experimental studies indicate that compression exceeding 50% of the dural sac cross-section significantly impairs venous drainage and axoplasmic transport, leading to intraneural oedema and conduction block [16]. Multilevel compression or pre-existing canal stenosis further intensifies ischemia by simultaneously limiting arterial inflow and venous outflow [17]. Beyond mechanical and vascular compromise, disruption of axonal transport interferes with mitochondrial trafficking and energy delivery, resulting in cytoskeletal disorganisation and adenosine triphosphate depletion [18]. Mitochondrial dysfunction generates reactive oxygen species, promoting oxidative damage to neuronal membranes and myelin sheaths [19].

Neuroinflammatory pathways also play a critical role. Activated Schwann cells and macrophages release pro-inflammatory cytokines, including tumour necrosis factor-α (TNF-α), interleukin-1β (IL-1β), and interleukin-6 (IL-6), which increase vascular permeability, exacerbate oedema, and inhibit remyelination [20]. Persistent inflammation may explain ongoing neuropathic pain and incomplete recovery in some patients despite timely surgical decompression. Excitotoxic mechanisms further amplify neural injury. Excessive glutamate release during ischemia overstimulates NMDA receptors, causing intracellular calcium overload, mitochondrial failure, and caspase-mediated apoptosis [21]. Concurrent Schwann cell loss leads to segmental demyelination, reducing nerve conduction velocity and prolonging sensory deficits [22]. Emerging research highlights potential adjunctive neuroprotective strategies, including TNF-α inhibition, antioxidant therapy (e.g., N-acetylcysteine, melatonin), and anti-apoptotic agents, which may complement surgical decompression in the future [23,24].

Massive central lumbar disc herniation remains the most common cause of CES, accounting for approximately 45-55% of cases, most frequently at the L4-L5 and L5-S1 levels, which together constitute nearly 75% of lumbar disc herniations overall [4]. This predominance is primarily attributable to the high frequency of disc herniation at these levels, while pre-existing degenerative lumbar spondylosis and reduced canal reserve may further predispose patients to symptomatic neural compression. CES most commonly affects middle-aged adults and is characteristically associated with large central or paracentral disc herniations, causing severe canal compromise. CES secondary to disc herniation may present acutely with rapid neurological deterioration or develop insidiously in patients with underlying lumbar stenosis. Large midline herniations occupying more than two-thirds of the spinal canal are particularly associated with CES [25]. Genetic predispositions, including collagen IX polymorphisms and degenerative disc disease, may increase susceptibility, although their clinical relevance remains limited [26]. Traumatic CES typically results from high-energy mechanisms such as burst fractures, fracture-dislocations, or penetrating spinal injuries, where nerve roots are subjected to both compression and shear forces [27].

Postoperative or iatrogenic CES, though uncommon, carries significant clinical implications. Causes include postoperative epidural haematoma following decompression or instrumentation, complications of epidural anaesthesia, catheter misplacement, and excessive dural sac retraction leading to traction injury [28,29]. In addition to mechanical compression, CES may arise from non-mechanical or systemic conditions. Infective pathologies such as epidural abscesses and spinal tuberculosis can produce chronic CES, particularly in endemic regions, while neoplastic causes include both primary spinal tumours and metastatic lesions [30].

Discussion

Clinical Presentation

CES typically manifests with symptoms reflecting dysfunction of multiple lumbosacral nerve roots. The classic symptom triad comprises bladder and/or bowel disturbance, reduced perineal (saddle) sensation, and sexual dysfunction [31]. Additional features include bilateral or asymmetric sciatica and variable degrees of lower-limb weakness or areflexia. Importantly, CES may present in an incomplete or evolving form. Contemporary guidelines, therefore, stress that any “red flag” symptom-even if transient or partial-should prompt urgent evaluation and MRI imaging. Gardner et al. emphasised that early recognition and meticulous documentation are crucial, given that delayed diagnosis represents a major source of litigation in spinal practice [4].

Bladder involvement is the most diagnostically significant and prognostically important feature of CES [32]. Early manifestations include altered urinary sensation, reduced urge to void, weak stream, or difficulty initiating micturition. However, recognition of early bladder dysfunction may be delayed, as patients may void only a few times daily, allowing several hours to elapse before difficulty with micturition becomes clinically apparent. Advanced stages are characterised by painless urinary retention with overflow incontinence resulting from complete detrusor areflexia. CES-I often precedes full retention, with subtle perineal sensory changes or voiding difficulty appearing hours to days earlier. This highlights CES as a dynamic process that can progress from reversible ischemia to permanent neural injury if treatment is delayed. Consequently, clinicians should maintain a low threshold for imaging, particularly in patients with new-onset urinary symptoms following back pain or sciatica [33].

Atypical presentations may include unilateral sciatica with early bladder involvement (commonly seen in L5-S1 herniations), isolated painless urinary retention in elderly individuals, or combined upper and lower motor neuron signs when the conus medullaris is involved [34].

Diagnostic Pitfalls and Differential Diagnosis

Although anatomically adjacent, CES and conus medullaris syndrome (CMS) differ in clinical and physiological characteristics. The conus medullaris, terminating near T12-L1, contains upper motor neurons and parasympathetic nuclei, whereas the cauda equina consists of lower motor neuron fibers, including the preganglionic parasympathetic fibers that travel through the pelvic splanchnic nerves to mediate bladder, distal gastrointestinal, and reproductive organ function. CES typically presents with flaccid weakness, areflexia, and asymmetric sensory loss, while CMS is more often associated with spasticity, hyperreflexia, and symmetric perianal sensory deficits.

Recognizing this distinction aids pre-imaging localization and diagnostic accuracy. Other neurological mimics-including acute transverse myelitis, Guillain-Barré syndrome, and multiple sclerosis-should also be considered [35]. A common diagnostic pitfall is attributing urinary retention solely to CES without excluding urological obstruction, infection, medication effects (e.g., anticholinergics), or psychogenic causes [36].

Classification Systems

Tandon and Sankaran (1967) proposed an early clinical classification based on symptom onset and prior spinal history. Type 1 CES describes sudden onset without previous back pain; type 2 refers to acute bladder dysfunction in patients with chronic back pain or sciatica (the most common presentation); and type 3 represents slowly progressive CES associated with lumbar stenosis or degenerative disease, typically in older individuals [37]. While not widely used for treatment decisions, this system aids understanding of disease progression and diagnostic urgency.

A more widely accepted prognostic classification was introduced by Gleave and Macfarlane [38]. CES-I is defined by preserved bladder sensation and some voluntary control, albeit with altered voiding. CES-R is characterized by painless urinary retention, loss of bladder sensation, and complete sphincter denervation. This distinction strongly correlates with outcome, as progression to CES-R is associated with poor prognosis regardless of decompression timing. Recognition of CES-I, therefore, represents the critical window for intervention.

Diagnostic Pathways and Imaging

The diagnosis of CES is primarily clinical, based on recognition of red-flag symptoms and confirmation through focused neurological examination. Maintaining a low threshold for suspicion is essential, particularly in patients presenting with new-onset bladder or perineal symptoms in the setting of back pain or sciatica. Comprehensive documentation of perineal sensation, anal tone, lower-limb motor and reflex status, and bladder function, including post-void residual assessment, is essential for both clinical management and medico-legal protection [39,40]. MRI is the diagnostic gold standard, providing direct visualization of neural compression, differentiation between mechanical and non-mechanical causes, and guidance for surgical planning. T1- and T2-weighted sagittal and axial sequences are routinely employed. In postoperative cases, contrast-enhanced MRI helps distinguish scar tissue from residual or recurrent disc material [41].

When MRI is contraindicated or unavailable, CT myelography serves as an alternative, although its invasive nature and inferior soft-tissue resolution limit its utility [42]. Nevertheless, in resource-constrained settings where immediate MRI access may not be feasible, alternative investigations may still aid in timely diagnosis and decision-making. Ancillary investigations such as bladder scanning are valuable; a post-void residual volume exceeding 200 ml is highly suggestive of CES and warrants urgent imaging [43]. Urodynamic studies and neurophysiological tests have limited acute diagnostic value but may aid chronic assessment and medico-legal evaluation [44,45].

Management and Timing of Surgery

Urgent surgical decompression remains the cornerstone of CES management. The primary goals are to relieve neural compression, restore perfusion, and prevent irreversible ischemic injury [46]. Adequate decompression must allow full visualization of affected nerve roots and dural sac expansion, with stabilization performed when spinal instability is present. Minimally invasive and microscopic techniques may reduce tissue trauma and postoperative morbidity [47].

The optimal timing of surgery remains debated. Meta-analyses suggest that decompression within 48 hours is associated with improved bladder, motor, sensory, and sexual outcomes [48,49]. While some studies report no significant difference between surgery within 24 versus 24-48 hours [50], others, including Srikandarajah et al., demonstrate superior bladder recovery when CES-I is decompressed within 24 hours [51]. CES-I and CES-R likely represent points along a dynamic clinical continuum rather than distinct isolated entities, with neurological deficits evolving progressively over time. Accordingly, once the diagnosis of CES is established, urgent surgical decompression should be performed as early as feasible in the interest of neurological preservation and prevention of further deterioration. Although autonomic recovery may be less predictable once CES-R develops, early decompression may still provide meaningful benefits in terms of pain relief, neurological preservation, and limitation of further functional decline [52,53].

Preoperative management focuses on pain control without oversedation, neutral spinal positioning, bladder catheterization to prevent overdistension, and bowel care to maintain hygiene and comfort [54,55]. As bladder dysfunction represents a cardinal diagnostic feature of CES, unnecessary early catheterization should be avoided whenever clinically feasible until urinary retention or significant bladder dysfunction has been appropriately assessed and documented. Once confirmed, bladder catheterization may be required to prevent overdistension and secondary detrusor injury. Pressure ulcer prevention and early multidisciplinary involvement are essential [56]. The role of corticosteroids remains controversial, with no definitive consensus supporting routine use [57].

Long-Term Outcomes and Rehabilitation

Prognosis following CES is highly variable and influenced by the stage at presentation, duration of compression, severity of deficits, etiology, and patient-specific factors [58]. Motor recovery typically precedes sensory improvement, while bladder and bowel function demonstrate the poorest recovery rates, with satisfactory continence achieved in only 30-50% of patients even after early surgery [38,59].

Rehabilitation requires a multidisciplinary approach incorporating physiotherapy, urological management, bowel programs, pelvic-floor therapy, sexual counselling, and occupational therapy to support functional independence and vocational reintegration [36,60,61]. Psychological sequelae, including depression and anxiety, are common and significantly affect quality of life. Cognitive behavioral therapy, pain management programs, and peer support play a critical role in long-term adjustment [62,63].

Emerging therapies - including stem-cell approaches, neurotrophic factor delivery, functional electrical stimulation, and pharmacological neuroprotection - offer promise but remain investigational [64-68]. Structured follow-up with serial neurological, urological, and psychosocial assessment is essential to optimize outcomes and ensure continuity of care.

## Conclusions

CES remains a true neurosurgical emergency, requiring prompt recognition, urgent imaging, and timely decompression to prevent permanent neurological impairment. Massive lumbar disc herniation is the most common cause, but traumatic, neoplastic, infectious, and iatrogenic etiologies must also be considered. Outcomes are closely linked to the timing of intervention and the stage of disease at presentation, with markedly better recovery observed in patients treated in the incomplete phase. Long-term management extends beyond surgery, involving comprehensive rehabilitation and psychosocial support. Future advances that integrate translational neuroscience, neuroprotective strategies, and AI-assisted diagnostics may further improve outcomes in this challenging condition.
